# Radiological Patterns of Abdominal Tuberculosis With Microbiological Confirmation: A Multi-Modal Prospective Analysis

**DOI:** 10.7759/cureus.113555

**Published:** 2026-07-28

**Authors:** Roja Dwarampudi, Bharath Kakileti, Deborah Purushottam M, Chinappareddy Saikiran Reddy, Katluru Avinash Redyy, Vidavaluru Sasank Sai

**Affiliations:** 1 Department of Radiology, Konaseema Institute of Medical Sciences and Research Foundation (KIMS), Amalapuram, IND; 2 Department of Radiodiagnosis, Konaseema Institute of Medical Sciences and Research Foundation (KIMS), Amalapuram, IND; 3 Department of Microbiology, Konaseema Institute of Medical Sciences and Research Foundation (KIMS), Amalapuram, IND

**Keywords:** abdominal tuberculosis, acid-fast bacilli, bowel involvement, cbnaat, diagnostic correlation, lymphadenopathy, multi-compartment disease, pcr, peritoneal tuberculosis, radiology

## Abstract

Objective: This study primarily aimed to evaluate the association between radiological patterns of abdominal tuberculosis (TB) and microbiological positivity using acid-fast bacillus (AFB) smear microscopy, cartridge-based nucleic acid amplification tests (CBNAATs), and polymerase chain reaction (PCR). Secondary objectives included describing the radiological manifestations of abdominal TB and comparing the microbiological diagnostic yields of these techniques.

Methodology: A prospective study was conducted in patients with suspected abdominal TB who underwent cross-sectional imaging, including ultrasonography (USG), computed tomography (CT), or magnetic resonance imaging (MRI), along with microbiological testing. Radiological patterns were categorized based on the site of involvement, and imaging findings were correlated with results from PCR, CBNAAT, and AFB smear examinations.

Results: A total of 126 patients with suspected abdominal TB were included. Bowel involvement was the most common radiological finding (112/126, 88.9%), followed by peritoneal involvement (88/126, 69.8%) and lymph node involvement (81/126, 64.3%). PCR demonstrated the highest microbiological positivity (98/126, 77.8%), followed by the CBNAAT (82/126, 65.1%) and AFB smear microscopy (44/126, 34.9%). Multi-compartment disease was significantly associated with higher microbiological positivity for PCR (42/46, 91.3% vs. 56/80, 70.0%; p=0.0109) and CBNAAT (37/46, 80.4% vs. 45/80, 56.3%; p=0.0108), whereas AFB smear positivity did not differ significantly between the groups (21/46, 45.7% vs. 23/80, 28.8%; p=0.085).

Conclusion: Radiological assessment demonstrates characteristic patterns of abdominal TB that are significantly associated with microbiological positivity, particularly in patients with multi-compartment involvement. However, microbiological confirmation remains essential for establishing the diagnosis. Molecular diagnostic methods such as PCR and CBNAATs showed higher microbiological yields than conventional AFB smear. An integrated approach combining radiological assessment with microbiological testing may facilitate earlier diagnosis and appropriate clinical management.

## Introduction

Tuberculosis (TB) remains one of the most significant infectious diseases worldwide, with the World Health Organization (WHO) estimating approximately 10.6 million new cases and 1.3 million deaths annually [1]. While pulmonary TB accounts for the majority of cases, extrapulmonary TB (EPTB) constitutes nearly 15-20% of all TB presentations, with abdominal TB being one of the most common forms of EPTB. In high-burden countries such as India, abdominal TB continues to pose a substantial diagnostic challenge owing to its nonspecific clinical presentation, chronic course, and close resemblance to a broad spectrum of benign and malignant abdominal conditions [2].

Abdominal TB may involve the gastrointestinal tract, peritoneum, lymph nodes, and solid viscera, with bowel, peritoneal, and nodal involvement forming the classical triad of disease. Among these sites, the ileocecal region is most frequently affected because of its abundant lymphoid tissue, physiological stasis, and relatively narrow lumen [3]. Peritoneal TB may present in exudative, fibroadhesive, or dry forms and often mimics peritoneal carcinomatosis. Lymph node involvement typically manifests as necrotic or conglomerated nodes, which can resemble lymphoma or metastatic disease [4].

Radiological imaging modalities, including ultrasonography (USG), computed tomography (CT), and magnetic resonance imaging (MRI), play a pivotal role in raising suspicion for abdominal TB and in delineating the extent and distribution of disease. Common imaging findings include bowel wall thickening, luminal strictures, ascites with fine internal septations, and necrotic lymphadenopathy. However, imaging findings alone are insufficient for a definitive diagnosis, as these features may overlap considerably with other inflammatory and neoplastic conditions [5].

Microbiological confirmation remains the diagnostic gold standard. Conventional Ziehl-Neelsen staining for acid-fast bacilli (AFB) is inexpensive and widely available but has limited sensitivity in paucibacillary extrapulmonary disease. Culture techniques improve diagnostic yield but are time-consuming and may delay treatment initiation [6]. In recent years, molecular diagnostic methods such as polymerase chain reaction (PCR) and cartridge-based nucleic acid amplification tests (CBNAATs), such as GeneXpert, have significantly advanced TB diagnostics by enabling rapid, sensitive, and specific detection. PCR enables identification of Mycobacterium tuberculosis DNA even in small quantities, while CBNAATs additionally provide information on rifampicin resistance, which is critical in the context of increasing multidrug-resistant TB [7].

Despite these advances, limited literature exists correlating radiological patterns of abdominal TB with multiple microbiological diagnostic modalities. Most available studies focus either on imaging characteristics or on individual diagnostic techniques. A comprehensive approach integrating radiological features with conventional smear methods and advanced molecular diagnostics may enhance diagnostic accuracy and facilitate the timely initiation of therapy.

The primary endpoint of this study was to evaluate the association between radiological patterns of abdominal TB and microbiological positivity using AFB smear, CBNAATs, and PCR. Secondary endpoints included describing the radiological spectrum of bowel, peritoneal, and lymph node involvement and comparing the microbiological yields of the different diagnostic techniques. This approach was designed to determine whether specific radiological patterns, particularly multi-compartment involvement, were associated with higher microbiological confirmation rates.

## Materials and methods

Study design and setting

This prospective observational study was conducted at a tertiary care referral hospital in Amalapuram over five years, from January 2020 to December 2024. Institutional Ethics Committee approval was obtained under IEC No. IEC/CD/2023 before data analysis and manuscript preparation. The study used prospectively collected clinical and radiological data from patients managed during the study period (January 2020-December 2024), and all analyses were performed in accordance with institutional ethical approval and the Declaration of Helsinki.

Study population

Patients of all ages and both sexes were enrolled according to predefined inclusion and exclusion criteria. The inclusion criteria comprised patients with clinical suspicion of abdominal TB presenting with symptoms such as chronic abdominal pain, fever, weight loss, altered bowel habits, or ascites. In addition, patients were required to demonstrate radiological features suggestive of abdominal TB on ultrasonography, computed tomography, or magnetic resonance imaging, and to have at least one microbiological investigation, such as AFB smear, CBNAATs, or PCR, performed on peritoneal fluid, lymph node aspirates, or bowel tissue biopsy specimens.

Patients were excluded if they had a previously established diagnosis of abdominal malignancy, lymphoma, or Crohn’s disease confirmed on histopathological examination; had incomplete medical records or missing microbiological data; or had initiated anti-tubercular therapy before radiological evaluation.

Data collection

Demographic data, including age and sex, along with clinical presentation and relevant laboratory parameters, were retrieved from medical records. Imaging reports and available radiological images were independently reviewed by two senior radiologists who were blinded to microbiological results. Discrepancies in image interpretation were resolved by consensus.

Radiological assessment

Radiological findings were systematically categorized into three principal compartments: bowel, peritoneal, and lymph node involvement using predefined imaging criteria. Bowel involvement was assessed for ileocecal wall thickening (defined as bowel wall thickness >3 mm in adequately distended bowel loops), asymmetric or circumferential mural thickening, single or multiple strictures, a pulled-up cecum, and mass-like lesions mimicking carcinoma. Peritoneal involvement was evaluated based on the presence of free or loculated ascites, fine internal septations, smooth or nodular peritoneal thickening, and omental thickening or caking. Lymph node involvement was assessed by the presence of enlarged mesenteric or retroperitoneal lymph nodes (short-axis diameter ≥10 mm), central low attenuation with peripheral rim enhancement suggestive of caseating necrosis, and conglomerated or matted lymph nodes. USG was primarily used for the initial evaluation of abdominal pathology and ascites, CT served as the principal imaging modality for disease characterization, and MRI was performed in selected patients requiring further anatomical assessment or problem-solving.

Microbiological investigations

Microbiological data were obtained from institutional laboratory records. AFB smear microscopy was performed using Ziehl-Neelsen staining on peritoneal fluid, lymph node aspirates, or tissue biopsy samples. The CBNAAT was carried out using the GeneXpert platform for the detection of Mycobacterium tuberculosis DNA and the assessment of rifampicin resistance. PCR assays, including conventional or real-time PCR, were employed to detect specific M. tuberculosis gene sequences, depending on sample availability. 

Routine mycobacterial culture was not performed in all patients because of limited laboratory availability, prolonged turnaround time, and inadequate specimen volume in some cases. Therefore, microbiological confirmation was based on AFB smear microscopy, CBNAAT, and PCR, which represented the standard diagnostic approach at our institution during the study period.

Data analysis

Radiological findings were correlated with microbiological results to assess diagnostic associations. The sensitivity of individual microbiological tests was compared across different anatomical sites of involvement. Patients with microbiological positivity were classified as definitive TB cases, whereas those with negative microbiological results and characteristic clinical and radiological features were categorized as probable TB cases. All radiological examinations were independently reviewed by two senior radiologists experienced in abdominal imaging, who were blinded to the microbiological findings. In cases of disagreement, the images were reviewed jointly, and a consensus interpretation was reached. Standardized imaging criteria were applied throughout the study to ensure consistency in radiological interpretation. Because radiological findings constituted part of the inclusion criteria, the present study should be interpreted as an observational correlation study within a cohort of patients with suspected abdominal TB rather than a formal diagnostic accuracy study. Consequently, the analyses focused on associations between radiological patterns and microbiological positivity rather than estimation of diagnostic sensitivity or specificity.

Statistical analysis was performed using IBM SPSS Statistics for Windows, Version 26 (Released 2018; IBM Corp., Armonk, New York, United States). Categorical variables were summarized as frequencies and percentages and compared using Pearson’s Chi-square test. Pearson’s Chi-square test was selected because it was appropriate for assessing the association between categorical variables in the present study. A p-value <0.05 was considered statistically significant.

## Results

Table 1 presents the demographic and clinical characteristics of the 126 patients. The mean age was 37.4 ± 12.6 years. The cohort included 62 (49.2%) male patients and 64 (50.8%) female patients. The most frequent clinical feature was abdominal pain, observed in 98 (77.8%) patients, followed by fever in 82 (65%) patients, weight loss in 73 (57.9%) patients, altered bowel habits in 53 (42.1%) patients, and ascites in 42 (33.3%) patients.

**Table 1:** Demographic and clinical characteristics of patients (n=126) Data are presented as numbers (N) and percentages (%)

Parameter	Observation (%)
Mean age (years)	37.4 ± 12.6
Male:Female	62:64
Abdominal pain	98 (77.8%)
Fever	82 (65.0%)
Weight loss	73 (57.9%)
Altered bowel habits	53 (42.0%)
Ascites	42 (33.3%)

Table 2 summarizes the radiological findings by the site of involvement. Among the 51 patients with bowel involvement, ileocecal wall thickening was the most common finding, observed in 28 (22.2%) patients, followed by a pulled-up cecum in 14 (11.1%) patients and multiple short strictures in nine (7.1%) patients. Of the 44 patients with peritoneal involvement, free ascites was observed in 30 (23.8%) patients, septated or loculated ascites in eight (6.3%) patients, and omental caking or thickening in six (4.8%) patients. Among the 31 patients with lymph node involvement, rim-enhancing necrotic nodes were identified in 19 (15%) patients, matted or conglomerated nodes in seven (5.5%) patients, and isolated mesenteric nodes in five (4%) patients.

**Table 2:** Radiological patterns of abdominal tuberculosis Data are presented as numbers (N) and percentages (%)

Site of involvement	Radiological finding	Cases (%)
Bowel (n=51)	Ileocecal wall thickening	28 (22.2)
	Pulled-up cecum	14 (11.1)
	Multiple short strictures	9 (7.1)
Peritoneum (n=44)	Free ascites	30 (23.8)
	Septated/loculated ascites	8 (6.3)
	Omental caking/thickening	6 (4.8)
Lymph nodes (n=31)	Rim-enhancing necrotic nodes	19 (15.0)
	Matted conglomerated nodes	7 (5.5)
	Isolated mesenteric nodes	5 (4.0)

Table 3 summarizes the microbiological test results and mean turnaround times. AFB smear was positive in 44 (34.9%) patients, with results available within 24-48 hours. The CBNAAT detected Mycobacterium tuberculosis in 82 (65%) patients and had a turnaround time of less than 48 hours. PCR demonstrated the highest positivity, identifying TB in 98 (77.7%) patients, with results reported within 24-72 hours.

**Table 3:** Microbiological yield in abdominal tuberculosis AFB: acid-fast bacilli; CBNAAT: cartridge-based nucleic acid amplification test; PCR: polymerase chain reaction Data are presented as numbers (N) and percentages (%)

Test	Positive cases (%)	Mean turnaround time
AFB smear	44 (34.9%)	24–48 hours
CBNAAT	82 (65.0%)	<48 hours
PCR	98 (77.7%)	24–72 hours

Among patients with bowel-only involvement (n=51), AFB smear was positive in 14 (27.4%) patients, CBNAAT in 27 (52.9%) patients, and PCR in 36 (70.5%) patients. In patients with peritoneum-only involvement (n=44), AFB smear positivity was observed in 12 (27.2%), CBNAAT in 23 (52.2%), and PCR in 32 (72.7%). For lymph node-only involvement (n=31), nine (29%) patients were AFB-positive, 18 (58%) were CBNAAT-positive, and 24 (77.4%) were PCR-positive. In cases with multi-compartment involvement (n=46), microbiological yields were higher. A total of 21 (45.6%) patients were positive on AFB smear, 37 (80.4%) patients on CBNAAT, and 42 (91.3%) patients on PCR (Table 4).

**Table 4:** Radiology–microbiology correlation AFB: acid-fast bacilli; CBNAAT: cartridge-based nucleic acid amplification test; PCR: polymerase chain reaction Data are presented as numbers (N) and percentages (%)

Site involvement	AFB positive (%)	CBNAAT positive (%)	PCR positive (%)
Bowel only (n=51)	14 (27.4)	27 (52.9)	36 (70.5)
Peritoneum only (n=44)	12 (27.2)	23 (52.2)	32 (72.7)
Lymph nodes only (n=31)	9 (29.0)	18 (58.0)	24 (77.4)
Multi-compartment (n=46)	21 (45.6)	37 (80.4)	42 91.3)

Table 5 presents the chi-square analysis comparing microbiological positivity between multi-compartment and single-compartment involvement. For AFB smear, the association with multi-compartment disease was not statistically significant (χ² = 2.965, df = 1, p = 0.085). In contrast, both CBNAAT and PCR positivity were significantly higher in patients with multi-compartment involvement, with χ² values of 6.490 (df = 1, p = 0.0108) and 6.486 (df = 1, p = 0.0109), respectively. These findings indicate that molecular diagnostic yields increase significantly when multiple anatomical compartments are involved.

**Table 5:** Chi-square test of association (multi vs single compartment) χ² (Pearson): chi-square (Pearson’s chi-square test) AFB: acid-fast bacilli; CBNAAT: cartridge-based nucleic acid amplification test; PCR: polymerase chain reaction

Test	χ² (Pearson)	df	p-value
AFB	2.965	1	0.085
CBNAAT	6.490	1	0.0108
PCR	6.486	1	0.0109

Table 6 presents the relative risk of microbiological test positivity in patients with multi-compartment involvement compared with single-compartment disease. For AFB smear, positivity was 21/46 (45.7%) in the multi-compartment group versus 23/80 (28.8%) in the single-compartment group, corresponding to a relative risk (RR) of 1.59 (95% CI: 0.995-2.534). CBNAAT positivity was higher in the multi-compartment group at 37/46 (80.4%) compared with 45/80 (56.3%) in the single-compartment group, yielding an RR of 1.43 (95% CI: 1.125-1.818). PCR showed the highest positivity in multi-compartment cases (42/46; 91.3%) compared with single-compartment cases (56/80; 70.0%), with an RR of 1.30 (95% CI: 1.102-1.544). These results indicate that multi-compartment disease is associated with a significantly increased likelihood of microbiological detection, particularly with molecular methods.

**Table 6:** Relative risk (RR) of test positivity (multi vs single), with 95% CI AFB: acid-fast bacilli; CBNAAT: cartridge-based nucleic acid amplification test; PCR: polymerase chain reaction

Test	Positivity in multi (p1)	Positivity in single (p2)	Relative risk (multi/single)	95% CI (lower — upper)
AFB	21/46 = 0.4565	23/80 = 0.2875	1.588	0.995 – 2.534
CBNAAT	37/46 = 0.8043	45/80 = 0.5625	1.430	1.125 – 1.818
PCR	42/46 = 0.9130	56/80 = 0.7000	1.304	1.102 – 1.544

## Discussion

In this prospective cohort of 126 patients with suspected abdominal TB, the radiological spectrum and microbiological yield largely mirrored findings reported in previous literature, while also revealing context-specific observations relevant to high-burden settings. Patients with multi-compartment abdominal TB demonstrated significantly higher microbiological positivity, particularly with PCR and CBNAATs. This finding may reflect a greater bacillary burden and the increased likelihood of obtaining representative tissue samples from multiple involved anatomical sites, including necrotic lymph nodes and bowel lesions. Previous studies have similarly reported that tissue biopsies and lymph node aspirates provide higher microbiological yields than ascitic fluid alone, especially when molecular diagnostic techniques are used, thereby improving diagnostic confirmation in abdominal TB [8-15]. Our findings are consistent with these observations and further support the complementary role of radiological assessment in guiding targeted microbiological sampling. The ileocecal region was the most frequently involved bowel segment, with predominant imaging features including asymmetric wall thickening, a pulled-up cecum, and short-segment strictures. These findings are consistent with classical radiological descriptions of abdominal TB. Sharma et al. [6] identified ileocecal mural thickening with associated necrotic lymph nodes as hallmark CT features useful in differentiating TB from Crohn’s disease and malignancy. Similarly, Balthazar’s [7] early CT series documented circumferential thickening of the terminal ileum and cecum with accompanying pericecal lymphadenopathy as common manifestations.

Peritoneal involvement in the present study most often manifested as free or loculated ascites with fine internal septations and smooth peritoneal thickening, whereas omental caking was predominantly observed in advanced disease. These findings align with prior series and pictorial reviews describing the classical wet, dry, and fibrotic-fixed forms of peritoneal TB, and emphasizing that septated ascites with smooth peritoneal nodularity favors TB over peritoneal carcinomatosis [8-12].

A notable finding of this study was the progressive increase in microbiological positivity with the use of more sensitive diagnostic techniques, with positivity rates of 35% for AFB smear, 65% for CBNAAT, and 78% for PCR. This gradient reflects the paucibacillary nature of extrapulmonary TB, particularly in abdominal disease, where conventional smear microscopy is less sensitive. Molecular diagnostic methods, especially PCR-based assays, have therefore been shown to enhance detection substantially. Das et al. [13] demonstrated improved diagnostic performance for intestinal and peritoneal TB using multiplex PCR, particularly when combined with adjunctive modalities such as histopathology or adenosine deaminase testing.

Despite its widespread adoption, CBNAATs have demonstrated variable sensitivity for abdominal TB across studies. Several prospective Indian series have reported CBNAAT positivity rates of approximately 30-35% in abdominal specimens from mixed extrapulmonary TB cohorts. Systematic and narrative reviews consistently indicate that although GeneXpert has high specificity, its sensitivity for peritoneal and intestinal samples remains heterogeneous and is frequently inferior to targeted PCR assays or histopathological examination [8,13,14]. The higher CBNAAT yield observed in the present study may be attributed to greater use of tissue biopsies and lymph node aspirates rather than ascitic fluid alone, improved pre-analytical handling, potential use of GeneXpert Ultra cartridges, and a higher proportion of patients with multi-compartment disease, in whom bacillary load is likely increased.

PCR-based testing demonstrated the highest overall diagnostic yield in this cohort, supporting evidence that multiplex or nested PCR assays outperform single-target molecular tests in intestinal and peritoneal TB. Recent literature syntheses emphasize that diagnostic strategies integrating molecular assays with histopathology, culture, or biochemical markers provide the greatest sensitivity and overall diagnostic accuracy [14]. The present findings reinforce this concept, indicating that reliance on a single diagnostic modality may result in underdiagnosis in clinically and radiologically suggestive cases.

A crucial analytical observation was the significantly higher microbiological detection rate among patients with multi-compartment involvement, defined as disease affecting two or more anatomical compartments. Both CBNAATs and PCR positivity were considerably higher in this group, while a similar trend was observed for AFB smear microscopy, although statistical significance was not achieved. This association is clinically intuitive, as more extensive disease involving the bowel, peritoneum, and necrotic lymph nodes increases the likelihood of sampling bacilli-rich tissue. Prior reviews similarly emphasize that lymph node aspirates and tissue biopsies yield higher sensitivity than ascitic fluid alone, unless loculated collections or adjunctive molecular or biochemical tests are employed [13,14].

The CBNAAT remains a valuable diagnostic tool because of its rapid turnaround time and ability to detect rifampicin resistance, which has important therapeutic and public health implications. In the present study, rifampicin resistance was identified in nine patients, underscoring the clinical utility of CBNAATs. However, existing literature cautions against its use as a standalone diagnostic test for abdominal TB, as sensitivity is highly dependent on specimen type and bacillary load. Consequently, a negative CBNAAT result does not reliably exclude disease, particularly in cases of isolated peritoneal or bowel involvement. The improved performance of CBNAATs in multi-compartment disease and tissue-based samples observed in this study is consistent with these reports [13-15].

Collectively, the findings of this study support a pragmatic, multimodal diagnostic approach for suspected abdominal TB. Multi-compartment radiological involvement was significantly associated with higher microbiological positivity, particularly with PCR and CBNAATs. These findings suggest that radiological assessment may help identify appropriate sites for targeted microbiological sampling, while microbiological confirmation remains essential for establishing the diagnosis.

Limitations and strengths

This study has several limitations. First, although mycobacterial culture remains the conventional reference standard for the diagnosis of TB, it was not routinely performed in all patients because of limited laboratory availability, prolonged turnaround time, and variable specimen adequacy during the study period. Consequently, microbiological confirmation relied primarily on AFB smear microscopy, CBNAATs, and PCR, and the findings should therefore be interpreted as demonstrating associations between radiological patterns and microbiological positivity rather than formal diagnostic accuracy. Second, because radiological findings formed part of the inclusion criteria, the study may be subject to verification bias and should be regarded as an observational correlation study within a cohort of patients with suspected abdominal TB rather than a diagnostic accuracy study. Third, this was a single-center study, which may limit the generalizability of the findings. In addition, not all patients underwent identical microbiological investigations, reflecting routine clinical practice.

Despite these limitations, the study has several strengths. Its prospective design, relatively large cohort, blinded independent radiological assessment, and integrated evaluation of radiological findings with multiple microbiological techniques provide clinically relevant evidence regarding the relationship between imaging patterns and microbiological confirmation in abdominal TB. Comparing microbiological yields by anatomical compartmental involvement may also help clinicians select appropriate sites for diagnostic sampling.

Future directions

Future prospective studies should standardize specimen types and pre-analytic protocols, such as tissue versus fluid processing and centrifugation techniques. Head-to-head comparisons of GeneXpert Ultra versus older cartridges and multiplex PCR, ideally using culture or histopathology as composite reference standards, are warranted. Additionally, the evaluation of novel assays, including cell-free M. tuberculosis DNA, immuno-PCR, and IGRA performed on peritoneal cells, appears promising based on recent reviews and deserves further clinical validation.

## Conclusions

The radiological patterns observed in our cohort were consistent with the established imaging spectrum of abdominal TB involving the bowel, peritoneum, and lymph nodes. Multi-compartment radiological involvement was significantly associated with higher microbiological positivity, particularly with PCR and CBNAATs. These findings support the complementary role of radiological assessment in identifying disease extent and guiding appropriate sites for microbiological sampling. However, microbiological confirmation remains essential for establishing the diagnosis. Further multicenter studies incorporating mycobacterial culture and standardized diagnostic protocols are warranted to validate these findings.
